# Beehives possess their own distinct microbiomes

**DOI:** 10.1186/s40793-023-00460-6

**Published:** 2023-01-09

**Authors:** Lorenzo A. Santorelli, Toby Wilkinson, Ronke Abdulmalik, Yuma Rai, Christopher J. Creevey, Sharon Huws, Jorge Gutierrez-Merino

**Affiliations:** 1 School of Biosciences, University of Surrey, Guildford, GU2 7XH UK; 2 The Roslin Institute, University of Edinburgh, Midlothian, EH25 9RG UK; 3 School of Biological Sciences, Institute for Global Food Security, Queen’s University Belfast, Belfast, BT9 5DL UK; 4 Present Address: Institute for Molecular Infection Biology, University of Würzburg, Würzburg, Germany

**Keywords:** Beehives, Apiary, Microbiome, Pollen, Honey, Honeybees, Gut commensals

## Abstract

**Background:**

Honeybees use plant material to manufacture their own food. These insect pollinators visit flowers repeatedly to collect nectar and pollen, which are shared with other hive bees to produce honey and beebread. While producing these products, beehives accumulate a considerable number of microbes, including bacteria that derive from plants and different parts of the honeybees’ body. Whether bacteria form similar communities amongst beehives, even if located in close proximity, is an ecologically important question that has been addressed in this study. Specific ecological factors such as the surrounding environment and the beekeeping methods used can shape the microbiome of the beehive as a whole, and eventually influence the health of the honeybees and their ecosystem.

**Results:**

We conducted 16S rRNA meta-taxonomic analysis on honey and beebread samples that were collected from 15 apiaries in the southeast of England to quantify the bacteria associated with different beehives. We observed that honeybee products carry a significant variety of bacterial groups that comprise bee commensals, environmental bacteria and symbionts and pathogens of plants and animals. Remarkably, this bacterial diversity differs not only amongst apiaries, but also between the beehives of the same apiary. In particular, the levels of the bee commensals varied significantly, and their fluctuations correlated with the presence of different environmental bacteria and various apiculture practices.

**Conclusions:**

Our results show that every hive possesses their own distinct microbiome and that this very defined fingerprint is affected by multiple factors such as the nectar and pollen gathered from local plants, the management of the apiaries and the bacterial communities living around the beehives. Based on our findings, we suggest that the microbiome of beehives could be used as a valuable biosensor informing of the health of the honeybees and their surrounding environment.

**Supplementary Information:**

The online version contains supplementary material available at 10.1186/s40793-023-00460-6.

## Introduction

Honeybees (*Apis mellifera*) use plant material to produce honey and beebread [1, 2]. Honey is made in the stomach of the adult workers, where the nectar collected from flowers is digested before regurgitation. Beebread is the collected pollen mixed with the young workers’ saliva. Both products are then further processed by microbes, including fermentative bacteria and yeasts that are thought to be involved in the crucial step of preservation [2–5]. Whether these microbes derive from the bees, or the environment is a very intriguing question that the scientific community has only addressed very recently [6, 7].

Recent studies have reported that the composition of the microbial community found in honey is dependent on the variety of floral nectars used by the bees [2, 8]. The nectar seems to contribute more significantly to species richness and microbial abundance than the honeybee gut [9, 10]. This microbial divergence is even more obvious in the beebread, where most of the microbes present in the pollen originates from the soil and phyllosphere [3, 11]. Furthermore, the microbes of nectar and pollen can be transferred by bees from plant to plant, from (or to) other insects and pollinators, and also shared with house bees within the same beehive, including beneficial bacteria [5, 12, 13] and pathogens [14, 15]. To date, we know that bees may accumulate a significant variety of different bacteria in their beehives; however, most of the studies have employed isolated beehives, primarily focusing on the microbiome of the pollen (or nectar) or the bee [3, 6, 8, 10]. No previous investigations have delved into the bacterial communities present in different beehives and how their fluctuations mirror the environment where honeybees forage and live.

In this study we have used samples of honey and beebread from beehives representing same or different apiaries to determine their bacterial profile. We postulate that the microbiome of beehives reflects the specific ecosystem where they are located, including the beekeeping methods used, and that honeybee products can be used as pooled samples to elucidate the bacterial species present in that ecosystem.

## Methods

### Experimental design, sampling, and DNA extraction

To test our hypothesis, we used 16S rDNA metataxonomy to characterise and compare the bacterial diversity present in samples of honey and beebread (referred as to pollen henceforward) that were collected from 15 apiaries in southeast England. Sample collection took place between mid-June and mid-August and targeted several habitats and soils in 4 different counties, as well as different beehives, some of which were located within the same apiary (same postcode) (see sampling in the Additional file 1: Excel File). Samples were collected directly from honeycomb frames using sterile swab tubes for pollen and containers for honey from which ten grams of each were put into sterile tubes that were immersed in liquid nitrogen and stored at − 80 °C. To ensure full representation of the whole beehive, samples were collected from different parts of the honeycomb. One hundred milligrams of the frozen samples were then ground manually using a mortar and pestle under sterile conditions and DNA was extracted using the BIO101 FastDNA® SPIN Kit for Soil in conjunction with a FastPrep® cell disrupter instrument (Bio101, ThermoSavant, Qbiogene) as we have previously reported [16]. We know that this kit results in enhanced extraction of DNA from both Gram-positive and Gram-negative bacteria, and therefore a realistic representation of complex microbial environments.

### 16S rRNA gene sequencing and taxonomy

DNA was quantified and quality-assured with a Thermo Scientific™ Nanodrop, and sequenced using the Ion Torrent PGM sequencer as previously described [17]. The sequencing process targeted the V1–V2 variable region of the bacterial 16S rRNA gene as the length of this region match with the coverage capacity of the sequencer. The V1–V2 amplicons were generated in triplicates, pooled to minimize the effect of PCR bias, and subjected to sequencing using the Ion PGM Template OT2 400 and Ion PGM Hi-Q Sequencing kits (Life Technologies Ltd, Paisley, UK). In total, we sequenced 39 samples from the 15 apiaries, including 24 honey and 15 pollen samples representing at least 1 beehive from each apiary and, in some cases, between 1 and 4 beehives from the same apiary (see sampling in the Additional file 1: Excel File). Therefore, sample IDs were designated with a number (1–15) to identify the apiary, followed by H or P and then A, B, C or D to indicate the product -honey or pollen- and the different hives of the apiary, respectively. Samples where A, B, C or D is not indicated correspond to apiaries with only 1 beehive. Following sequencing we used The CD-HIT-OTU pipeline to remove low quality sequences, pyrosequencing errors and chimeras [18], with the resulting sequences clustered into Operational Taxonomic Units (OTUs) at 97% identity. OTUs were then taxonomically classified down to genus rank against the Greengenes 16S rRNA gene database (13.5) using MOTHUR [19].

### Statistical analysis

We performed calculations of alpha and beta diversity at genus level using the phyloseq Bioconductor package in R [20]. Alpha diversity was calculated to assess and compare the richness and evenness between apiaries and samples of honey and pollen based on the observed OTUs and the Inverse Simpson (InvSimpon) index. Different OTUS were converted to percentage of total reads and subjected to ANOVA with Tukey–Kramer post-hoc analysis for multiple comparisons with a confidence level of at least 95% (*p* < 0.05) using GenStat [21]. Results were illustrated using box and whisker plots. For the beta diversity calculations, we employed distance matrices of Jaccard and Bray–Curtis to identify compositional dissimilarities amongst apiaries based on the presence/absence and abundance of microbial communities, respectively. These dissimilarities were visualized on Principal Coordinates Analysis (PCoA) plots, and then analysed using permutation tests for Adonis (Permanova) and homogeneity of multivariate dispersions (Betadispersion) followed by Tukey–Kramer multiple comparisons with a confidence level of at least 95% (*p* < 0.05). Permanova and Betadispersion let us confirm significant differences in the composition and spread of the bacterial communities not only between apiaries but also between hives within the same apiary.

### Taxonomical analysis

To complement the alpha and beta diversity analysis described above, we used ClustVis [22] to cluster the OTUs from all the beehives based on the type of sample (honey and pollen) and the soil habitat. The soil types were identified using the postcode on the Cranfield University Soil and Agrifood Institute Soilscapes tool (http://www.landis.org.uk/soilscapes/). Despite being in different locations (postcodes) some apiaries share the same habitat and potentially a similar ecosystem around them, introducing a supplementary variable that could help group samples. The resulting clusters were visualized at phylum, class and order level using a heatmap. The identification of the OTUs at species level was finally performed by carrying out a BLASTN search of the representative sequences of each OTU against the NR database. A match was considered significant if it had greater or equal to 98% sequence identity and 100% coverage of the query sequence. Species that passed these filters were then classified as either bee symbionts, invertebrate symbionts, vertebrate symbionts, environmental bacteria, or pathogens by reference to the NCBI scientific literature.

## Results and discussion

### Sequencing data and analysis

The sequencing of the V1-V2 amplicons from the 39 samples generated 2.2 million reads, with an average of 57,500 reads/sample and length of 300 bp, and a total of 90 potential OTUs (see OTU’s raw data in the Additional file 1: Excel File). The read coverage was nearly 100% for all samples and, on average, 36% (min < 1%; max 99%) of the reads derived from each sample were non-bacterial, generally representing matches to chloroplasts or mitochondria of plants. The details of the target and off target OTUs are also indicated in the Additional file 1: Excel file. Although rarefaction curves showed that the sequencing depths of the 39 samples were adequate to maintain all of them in our analyses (Additional file 2: Fig. S1), we decided to exclude the OTUs that were only classified as plant as well as those with a bacterial identity % less than 97 and a very low confidence at phylum level (highlighted in red within the OUT’s raw data). Three pollen samples (6P; 10PA and 15P) with fewer than 2000 reads from OTUs classified as bacteria but containing very high levels of off-target matches were also ruled out. This resulted in 74 OTUs and 36 samples with an average of 29,868 reads per sample (see OUT’s normalized data in the Additional file 1: Excel File), from which all OTU counts were scaled to the minimum sample size for normalization and subsequent comparative analysis. The sequences of the initial 39 samples are available in the NCBI database under bioproject number PRJEB45401.

### Bacterial diversity in beehive samples

**Fig. 1:**
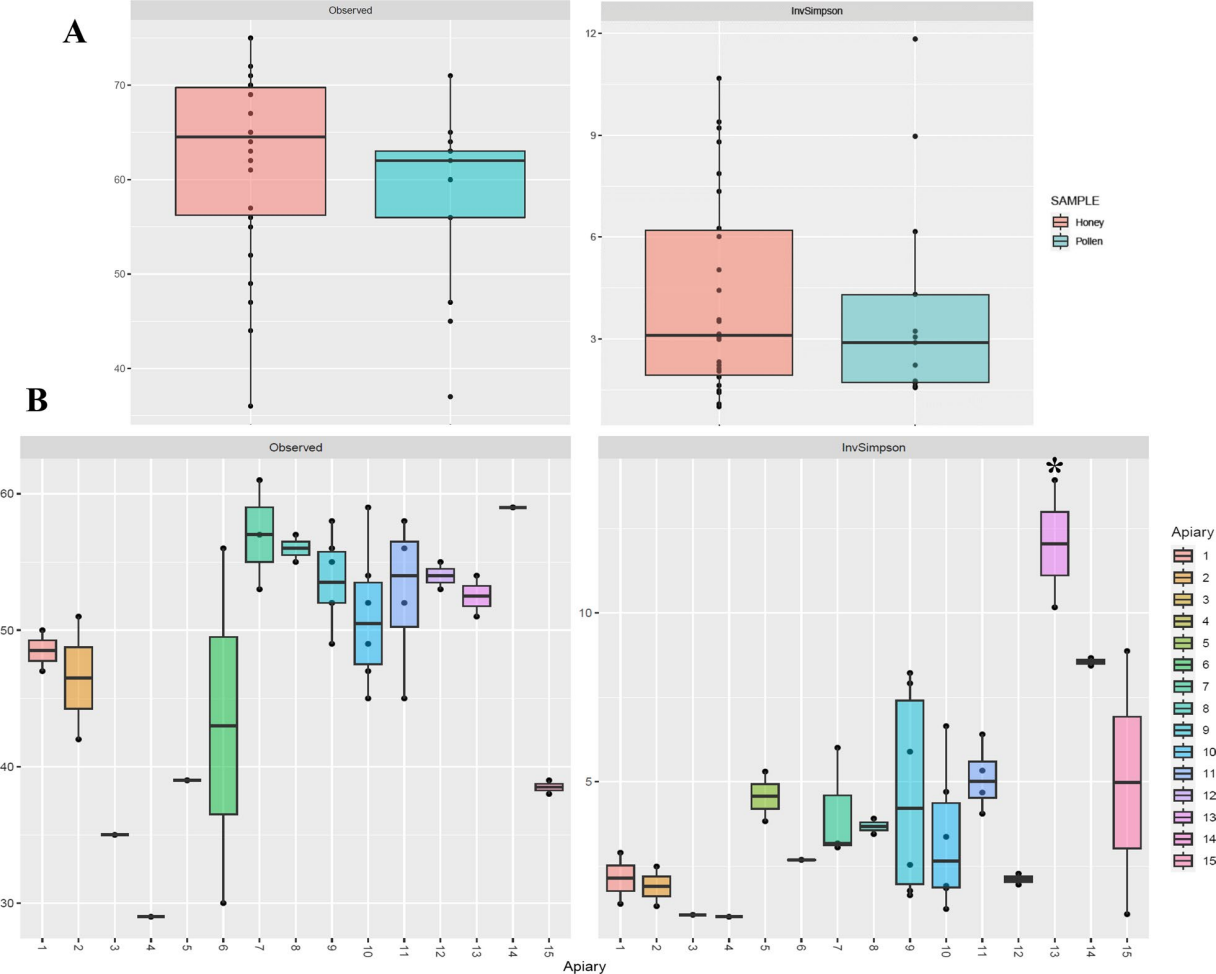
The observed-OTUs richness and inverse Simpson index of bacterial communities found in beehives and grouped by the type of sample (**A**) and different apiaries (**B**). Tukey–Kramer post-hoc analysis was carried out for multiple comparisons (*, *p* < 0.05)

The variety of bacterial communities present in all samples was first estimated using the two following alpha diversity measures: observed OTUs and InvSimpson index (Fig. 1). When samples were grouped by the type of honeybee product, no significant differences were observed between honey and pollen samples, either in terms of richness or evenness (Fig. 1A). Similar results were obtained using the apiaries as a grouping factor (Fig. 1B), specially with regards to the abundance of OTUs. The number of OTUs identified in all samples was not significantly different amongst the apiaries. However, estimations with the InvSimpon index revealed that apiary 13 display the highest diversity. With the exception of apiaries 5, 11, 14 and 15, the relative abundance of OTUs found in apiary 13 is significantly higher than that of the remainder apiaries. This first evidence of difference between apiaries led us to investigate the dissimilarities between the type of bacterial communities present in the samples.

**Fig. 2:**
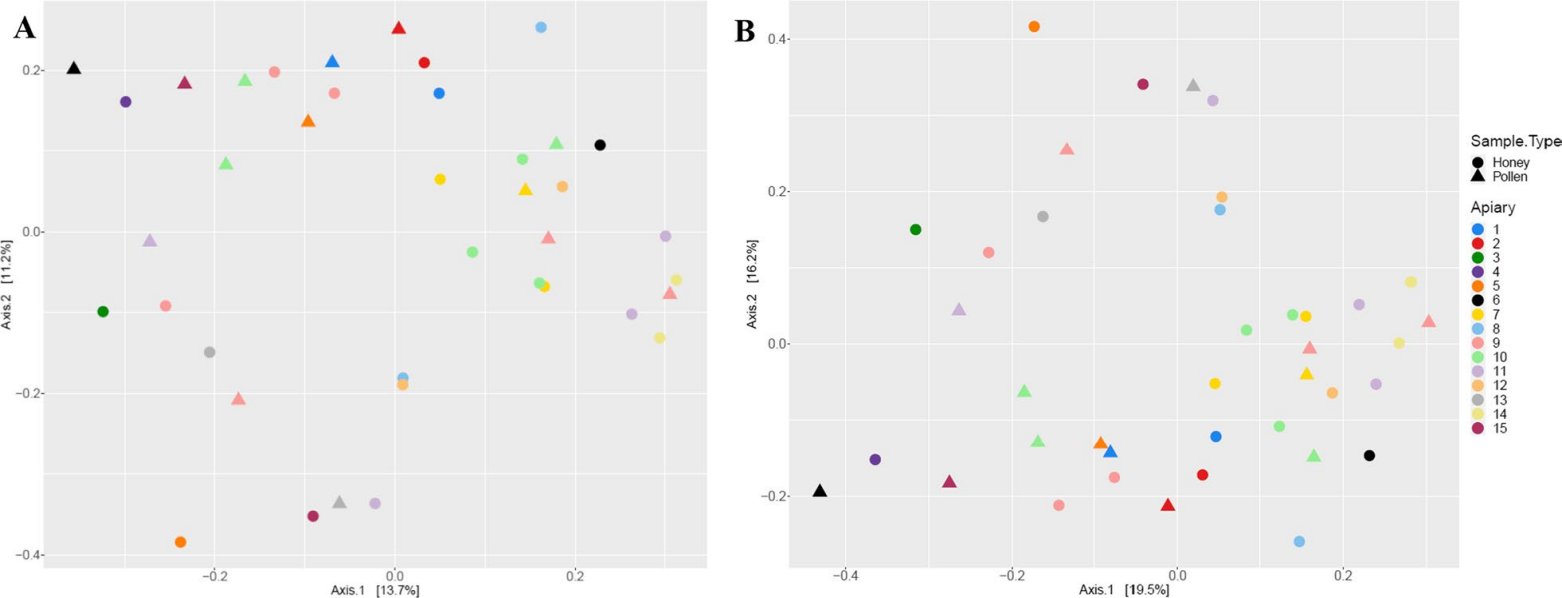
PCoA plots of the Jaccard (A) and Bray–Curtis (B) dissimilarities for bacterial communities (OTUs) found in samples of honey and pollen that were collected from the 15 different apiaries selected in this study. Samples with the same colour derive from the same apiary

To visualize the distance in the bacterial OTUs found in honey and pollen samples isolated from different apiaries, we generated Principal Coordinates Analysis (PCoA) plots based on Jaccard and Bray–Curtis dissimilarity metrics (Fig. 2). The plots show that samples did not group by product type (honey vs pollen) or apiary. In fact, the beta diversity Adonis test confirmed this different compositional dissimilarity between the apiaries with a confidence level of higher than 99% (*p* = 0.0056). In terms of distribution, the differences were even significantly higher (*p* < 0.001), especially when the OTUs of apiaries 3, 4 and 14 were pairwise compared with those of 5, 6, 9, 10 and 11 (*p* < 0.05). In this respect, the different location of the apiaries could explain these variabilities; however, it is worth highlighting that apiaries 3, 4 and 11 are in close proximity, with approximately 4 miles of distance between the 3 of them. On average, honeybees forage distances no longer than 5 miles [23]. Furthermore, when those apiaries represented by more than 2 beehives were compared (9, 10 and 11), we also observed significant differences in their bacterial communities (*p* < 0.05). Taken together, our data suggests that there might be no consistent bacterial fingerprint for beehives, even when samples of honey and pollen are taken from the same apiary. These divergences were confirmed following a comprehensive taxonomical analysis described below.

### Taxonomical analysis

The classification of the OTUs into different taxonomic ranks resulted in the identification of 5 different phyla across samples, with Firmicutes and Proteobacteria the most abundant, for which the number of different classes and orders detected were 7 and 19, respectively (Additional file 1: Excel file and Additional file 2: Fig. S2). Sequences from Gammaproteobacteria and Bacilli were very frequent, and within these classes, the orders Pseudomonadales, Enterobacterales and Lactobacillales were the most predominant. Of particular note is the fact that seven unclassified bacterial communities, potentially derived from animal faeces and tissues, were found in nearly all samples, with a very high relative proportion in sample 13H. Moreover, chloroplast sequences were also identified within honey and pollen samples, likely as a result of the bacterial origin of this organelle (Additional file 1: Excel file). Recent studies have reported that plant chloroplast sequences are very prevalent on honeybee products and can help to determine the foraging patterns of the bees [24]. In this study we observed matches to chloroplasts belonged to different plant species, including *Adenophora stricta*, *Citrullus lanatus*, *Fagus sylvatica*, *Quercus fenchengensis*, *Raphanus sativus* and *Salix paraflabellaris* (“Off target” OTUs in the Additional file 1: Excel file). However, it should be noted that 16S rDNA techniques do not give the best resolution for distinguishing different plants, with the RuBisCo large subunit (*rbcL*) and maturase K (*matK*) genes being better biomarkers [25].

**Fig. 3:**
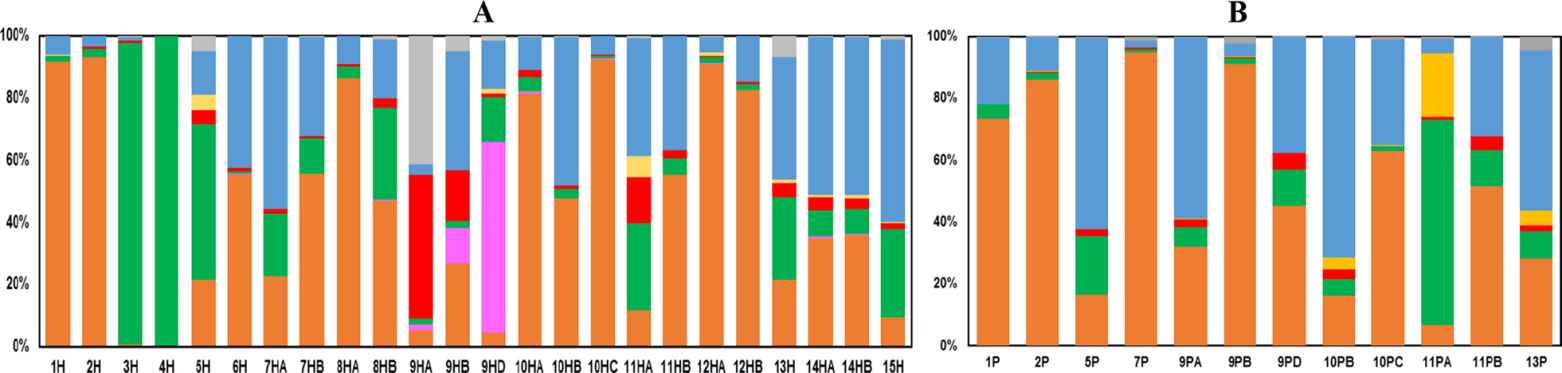
Bacterial communities of bee symbionts (orange), invertebrate symbionts (pink), vertebrate symbionts (yellow), environmental bacteria (green), and pathogens (red) found in honey (**A**) and pollen (**B**) samples. Other bacteria from which only the genus was identified are indicated in blue, while those unclassified are represented in grey. The species-level analysis of the identified OTUs revealed: (i) Bee symbionts (orange) isolated from honey, pollen and honeybees, including *Arsenophonus nasoniae*, *Bartonella apis*, *Bombilactobacillus mellis*, *Frischella perrara*, *Gilliamella apicola*, *Lactobacillus kunkeei*, *L. helsingborgensis*, *L. apis*, *Parasaccharibacter apium*, *Snodgrassella alvi*, and *Spiroplasma melliferum*; (ii) Invertebrate symbionts (pink) found in other insects and nematods, including *Commensalibacter intestine*, *Moraxella osloensis*, *Photorhabdus kayaii* and *Serratia symbiotica*; (iii) Vertebrate symbionts (yellow) found in the skin and gut of birds, mammals and humans, including *Acinetobacter pullicarnis*, *Haemophilus parainfluenzae*, *Lactobacillus salivarius*, and *Microbacterium hominis*; (iv) Environmental bacteria (green) found in water, soil, plants, seeds, fruits, food and animal faeces, some of which may cause infections in plants and animals, such as *Acinetobacter boissieri*, *A. chinensis*, *A. junii*, *Bacillus thuringiensis*, *Brevundimonas diminuta*, *B. mediterranea*, *Burkholderia cepacia*, *Cutibacterium acnes*, *Fructobacillus fructosus*, *F. tropaeoli*, *Lactococcus lactis*, *Leuconostoc mesenteroides*, *Methyloversatilis discipulorum*, *Neokomagataea tanensis*, *Pantoea vagans*, *P. agglomerans*, *Pelomonas puraquae*, *Pseudomonas fluorescens*, *P. graminis*, and *Zymobacter palmae*; (v) Pathogens (red) that cause diseases in plants, animals and humans, including *Enterococcus faecalis*, *Lonsdalea britannica*, *Pseudomonas syringae*, *Staphylococcus aureus*, *Xanthomonas campestris*, and *Yersinia mollaretii*; and (vi) other bacteria (blue) representing vertebrate symbionts and environmental bacteria, including *Acinetobacter*, *Erwinia*, *Fibrobacter*, *Mycoplasma*, *Pantoea*, *Prevotella*, *Ralstonia*, and *Undibacterium*

The clustering of OTUs at phylum, class and order level across the different samples and the various soil type habitats of the apiaries gave us, once again, a very undefined bacterial profile amongst the beehives (Additional file 2: Fig. S2). Samples of honey and pollen did not group at all, and although more similarity was observed using the soil variable, especially when samples derive from the same apiary, instances of variability were confirmed amongst the beehive samples, with no clear clusters grouped by soil-type habitat. To delve into the reasons of this irregular microbiome structure, we carried out a further OTU analysis at lower taxonomical levels. This analysis revealed the presence of 40 genera (Additional file 1: Excel file), from which we identified 44 different species representing different bacterial communities as referred to their ecological features (Fig. 3). Although most of the samples were dominated by bee symbionts and environmental bacteria (Fig. 3), we also detected symbionts of invertebrates and vertebrates, as well as potential pathogens of plants and humans such as *Enterococcus faecalis*, *Lonsdalea britannica*, *Pseudomonas syringae*, *Staphylococcus aureus*, *Xanthomonas campestris* and *Yersinia mollaretii* [26–28]. Similar to the lack of microbiome consistency discussed above, the abundance and distribution of OTUs within the different bacterial communities varied amongst the different beehive samples, with no clear core microbiome defining honey and pollen and the different apiaries that the samples represent (Fig. 3 and Additional file 1: Excel file). The only few species that were detected in all samples of honey and pollen included the plant endophyte *Cutibacterium acnes* [29] and the two bee symbionts *Lactobacillus kunkeei* and *Parasaccharibacter apium* [30]. Furthermore, we observed some contradictions to the general agreement that pollen carries more environmental bacteria than bee commensals, when compared to honey, and vice versa [9, 11]. For instance, the bee symbionts *Arsenophonus nasoni*ae, *Gilliamella apicola*, *Lactobacillus apis*, and *Snodgrassella alvi* [31] were more frequently found in our pollen samples, while the plant and water associated bacteria *Lactococcus lactis* [32], *Pelomonas puraquae* [33] and *Pseudomonas graminis* [34] showed a higher prevalence in honey. As expected, the number of honey samples populated by bacteria previously found in nectar, such as *Acinetobacter boissieri* [35] and *Fructobacillus fructosus* [36], was higher than that of pollen.

### Bacterial community of bee symbionts

Finally, we investigated why the community of bee symbionts fluctuate amongst the beehives (Fig. 3) and whether those fluctuations may be dependent on the presence and abundance of certain environmental bacteria and the apiculture methods used by the beekeepers. To answer these questions, we took advantage of the sequencing data from the 24 honey samples to obtain the total percentage of bee symbionts and environmental bacteria present in each of the samples. We also calculated the relative proportion of the different species and families found within both groups of bacteria. As indicated in Table 1, there was a clear correlation between the abundance of symbiotic and environmental bacteria and the use of antimicrobials in the beehives. On the one hand, the microbiome of apiaries that did not receive any antimicrobial treatment (e.g. amitraz, fumidil B, oxalic/formic acid and/or thymol), has an overrepresentation of the very well know honeybee gut commensal *L*. *kunkeei* (samples from apiaries 1, 2, 8 and 12) and a low percentage of bacteria commonly found in plants, including species of the *Erwiniacea* (*Pantoea agglomerans* and *P. vagans*) and *Pseudomonadaceae* (*Pseudomonas fluorescens* and *P. graminis*). In contrast, beehives exposed to antimicrobial treatments due to previous infestations and/or infections (Chalkbrood, *Nosema apis*, sacbrood, *Varroa*, and/or wax moth), showed a much lower percentage of symbiotic bacteria, but a more diverse community comprising not only *A. kunkeei* but also *Parasaccharibacter apium*, *Arsenophonus nasoniae*, *Serratia symbiotica* and *Gilliamella apicola* (samples from apiaries 7, 9, 11, 14, 15). This reduced level of bee commensals shifted significantly in favour of some environmental bacteria belonging to the families of *Propionibacteriaceae* (*Cutibacterium acnes*), *Lactobacillaceae* (*Fructobacillus fructosus*, *F. tropaeola*, *Lactobacillus salivarius*, *Leuconostoc mesenteroides*) and *Streptococcaceae* (*Lactococcus lactis*). In beehives where antimicrobial treatments were used as a prophylactic measure, the abundance of bee symbionts ranged from high to moderate or even low (samples from apiaries 5, 10, 13), but the structure of the bacterial communities was similar to that of the beehives suffering from infestations and/or infections and subsequent antimicrobial therapies.

**Table 1:** The percentage of bee symbionts and environmental bacteria and their corresponding species and families that were detected in honey samples representing 24 beehives where different antimicrobials were used as a prophylactic measure or therapeutic treatment due to previous infestations or infections

Sample	Bacterial communities				AM	INF
	Symbionts	Species of symbionts (relative %)	Environm	Families of environmental bacteria (relative %)		
1H	92	*L. kunkeei* (97)	8	*Erwiniaceae* (88), *Pseudomonadaceae* (10)	−	−
2H	93	*L. kunkeei* (97.4)	9	*Erwiniaceae* (60.4), *Pseudomonadaceae* (37)	−	−
3H	1	*P. apium* (58.5), *L. kunkeei* (39.2)	97	*Bacillaceae* (99.2)	−	−
4H	0.5	*L. kunkeei* (58.5), *A. nasoniae* (20.1), *P. apium* (12.5)	99	*Bacillaceae* (100)	−	−
5H	22	*P. apium* (88), *L. kunkeei* (8.8)	64	*Streptococcaceae* (65.8), *Propionibacteriaceae* (30.4)	+	−
6H	55	*L. kunkeei* (92.3), *P. apium* (6.5)	43	*Erwiniaceae* (85.4), *Bacillaceae* (8.6)	−	−
7HA	23	*L. kunkeei* (55.7), *A. nasoniae* (28), *P. apium* (10.3)	76	*Lactobacillaceae* (47), *Halomonadaceae* (36), *Propionibacteriaceae* (13)	+	+
7HB	56	*A. nasoniae* (97.3)	43	*Erwiniaceae* (55.4), *Propionibacteriaceae* (27.8)	+	+
8HA	86	*L. kunkeei* (57.3), *A. nasoniae* (24.3), *P. apium* (17.9)	13	*Erwiniaceae* (78), *Bacillaceae* (9)	−	−
8HB	47	*L. kunkeei* (84.2), *P. apium* (8)	49	*Propionibacteriaceae* (56), *Erwiniaceae* (23), *Streptococcaceae* (18)	−	−
9HA	5	*A. nasoniae* (32.2), *S. symbiotica* (29), *P. apium* (11.4)	5	*Erwiniaceae* (65.9), *Propionibacteriaceae* (28.4)	+	+
9HB	27	*A. nasoniae* (39.5), *S. symbiotica* (29.1), *P. apium* (5)	40	*Erwiniaceae* (94.1), *Lactobacillaceae* (3.1), *Propionibacteriaceae* (1.5)	+	+
9HD	5	*L. kunkeei* (40.2), *A. nasoniae* (21.9*), G. apicola* (15.6)	29	*Propionibacteriaceae* (75.9), *Erwiniaceae* (7.5), *Lactobacillaceae* (6)	+	+
10HA	81	*L. kunkeei* (90.9), *P. apium* (6.9)	16	*Lactobacillaceae* (34.8), *Pseudomonadaceae* (26), *Erwiniaceae* (20.8), *Propionibacteriaceae* (10.4)	+	−
10HB	48	*L. kunkeei* (91.4), *P. apium* (7.3)	51	*Erwiniaceae* (82.8), *Lactobacillaceae* (9.3)	+	−
10HC	92	*L. kunkeei* (72.9), *P. apium* (26.1)	7	*Erwiniaceae* (71.4), *Lactobacillaceae* (22.1)	+	−
11HA	12	*L. kunkeei* (60), *P. apium* (33), *A. nasoniae* (5.4)	66	*Propionibacteriaceae* (62.2), *Lactobacillaceae* (25), *Streptococcaceae* (7.4)	+	+
11HB	55	*L. kunkeei* (79.8), *P. apium* (17) *A. nasoniae* (2.7)	42	*Erwiniaceae* (48.4), *Lactobacillaceae* (40.8)	+	+
12HA	91	*L. kunkeei* (81), *B. apis* (6.2)	7	*Propionibacteriaceae* (50), *Erwiniaceae* (33), *Streptococcaceae* (10)	−	−
12HB	83	*L. kunkeei* (79.6), *A. nasoniae* (8.8)	17	*Erwiniaceae* (88), *Pseudomonadaceae* (7)	−	−
13H	21	*A. nasoniae* (61.8), *P. apium* (17.4), *L. kunkeei* (14.6)	66	*Pseudomonadaceae* (69), *Streptococcaceae* (13.2), *Erwiniaceae* (12.9), *Propionibacteriaceae* (9.1)	+	−
14HA	35	L*. kunkeei* (69.9), *P. apium* (16), *A. nasoniae* (8.4)	59	*Erwiniaceae* (72.4), *Lactobacillaceae* (12.8)	+	+
14HB	36	*L. kunkeei* (68.9), *P. apium* (16.6), *A. nasoniae* (9.2)	59	*Erwiniaceae* (73.1), *Lactobacillaceae* (13.3)	+	+
15H	9	*L. kunkeei* (81.5), *P. apium* (14.6), *A. nasoniae* (3.3)	77	*Streptococcaceae* (75.5), *Propionibacteriaceae* (14.7)	+	+

The relative % of symbionts and environmental bacteria refer to their total % and the species and families indicated in the table were selected from the groups established in Fig. 3 (symbionts of bees and insects in orange and pink, and environmental bacteria in green). The symbiotic species include *Lactobacillus kunkeei*, *Parasaccharibacter apium*, *Arsenophonus nasoniae*, *Serratia symbiotica*, *Gilliamella apicola*, and *Bartonella apis*, while families of environmental bacteria are represented by the following species: *Bacillus thuringiensis* (*Bacillaceae*), *Cutibacterium acnes* (*Propionibacteriaceae*), *Fructobacillus fructosus*, *F. tropaeola*, *Lactobacillus salivarius*, *Leuconostoc mesenteroides* (*Lactobacillaceae*), *Lactococcus lactis* (*Streptococcaceae*), *Pantoea agglomerans*, *P. vagans* (Erwiniacea), *Pseudomonas fluorescens*, *P. graminis* (*Pseudomonadaceae*), *Zymobacter palmae* (*Halomonadaceae*). AM (Antimicrobials), INF (Infestation and/or infection)

Here, we have shown that changes in the microbiome of the beehive, in particular those affecting the bee symbionts, associate with specific apiculture methods. This finding contradicts a recent study reporting that different hive practices do not influence the bee gut microbiome [37]. However, we must consider that the authors of this study used preparations of whole bees and dissected bee samples instead of samples originated from beehives. On the other hand, our data indicate that the use of antimicrobials could be an underlying factor leading to more susceptibility to infestations or infections in the beehive. Previous studies have shown that some species of symbionts are more abundant in bees from pathogen-infected colonies [38, 39]. For instance, *L. kunkeei* antagonizes bee pathogens [40], and this is the main symbiotic species that we have found in antibiotic-free beehives. However, the eventual presence of other species such *Bartonella apis*, *Frischella perrara*, *G. apicola*, and *Snodgrasella alvi*, which have also been detected in our samples, may be crucial to maintaining the health of the colony [41]. Additionally, we have observed that the presence of certain environmental bacteria correlates with the levels of bee symbionts, suggesting the influence of microbes that, in principle, derive from plants, water or soil, in the conformation of the beehive microbiome. On this point, samples derived from apiaries 3, 4 and 6 are a clear example of how spore-forming bacteria, in particular *Bacillus thuringiensis* may outcompete symbiotic bacteria and overpopulate the beehive, as recently reported [42].

## Conclusions

Our exploratory study shows that honeybee products carry a significant diversity of bacterial species, particularly from the bees, plants and the environment; and also that there is an inconsistent microbial pattern, not only between honey and pollen, but also among samples collected from the same apiary and/or habitat. In agreement with very recent studies, we have confirmed that the beehive microbiome is defined by multiple environmental and ecological factors, such as the soil, habitat, local plants and bee forage [43, 44]; and most importantly, our results suggest, for the first time, that every beehive possesses their own distinct bacteria. Furthermore, we have also described that the presence of certain environmental bacteria seems to influence the levels at which the most prevalent bee symbionts are detected. Whether apiaries have received any antimicrobial treatment may also affect the type of bacteria present in the beehives. We thus postulate that the use of antibiotics and the incidental existence of some environmental bacteria in the beehive defines the prevalence and abundance of honeybee gut symbionts. Depending on how beneficial these symbionts are, they might then promote or jeopardize the health of the colony.

In conclusion, we propose that the DNA present in honey and pollen could inform us of microbial changes indicative of the health of the beehive ecosystem, including not only social bees and plants but also solitary bees and other animals living within that ecosystem. Further ecological studies that include a comprehensive monitoring on the beehive microbiome throughout different seasons and years as well as additional sampling of potential sources of bacteria from the environment would be important to verify the origins and dynamics of the microbial communities present in the beehives.

## Supplementary Information

**Additional file 1.** Sampling data, OTU's raw data, OTU's normalized data, offtarget OTU's raw counts and target and offtarget OTU's data.

**Additional file 2: Fig S1.** Rarefaction curves illustrating the sequencing depth of bacterial communities (identified OTUs) in the 39 samples of honey and pollen collected from the 15 apiaries of this study. Curves were generated in R using the package ggrare and plotted with ggplot2. **Fig S2.** Clustering of OTUs from our beehive samples provides a proportional representation of microbiomes at 3 taxonomical levels (phylum-class-order) across different samples of honey and pollen and soil type habitats. The IDs for the samples are indicated at the bottom, where the numbers designate the apiary (postcode location) and letters H or P denote the product type -honey or pollen, respectively. If H or P are followed by another letter (A-B-C-D) indicates different beehives within the same apiary. Different soils include: 5, Herb-rich chalk and limestone pastures, lime-rich deciduous woodlands; 6, Neutral and acid pastures and deciduous woodlands, acid communities such as bracken and gorse in the uplands; 7, Base-rich pastures and deciduous woodlands; 8, Wide range of pasture and woodland types; 14, Mostly lowland dry heath communities; 15, Mixed dry and wet lowland heath communities; 18, Grassland and arable some woodland; and 22: Arable grassland and woodland. The heatmap was generated using ClustVis.

## Data Availability

The sequencing data is available at https://www.ncbi.nlm.nih.gov/bioproject/ under bio-project number PRJEB45401.
