# Supplementary figures and images for: Beehives possess their own distinct microbiomes
Source: Environ Microbiome. 2023 Jan 9;18:1. doi: 10.1186/s40793-023-00460-6 (PMC9830898; doi:10.1186/s40793-023-00460-6)

## Slide 1
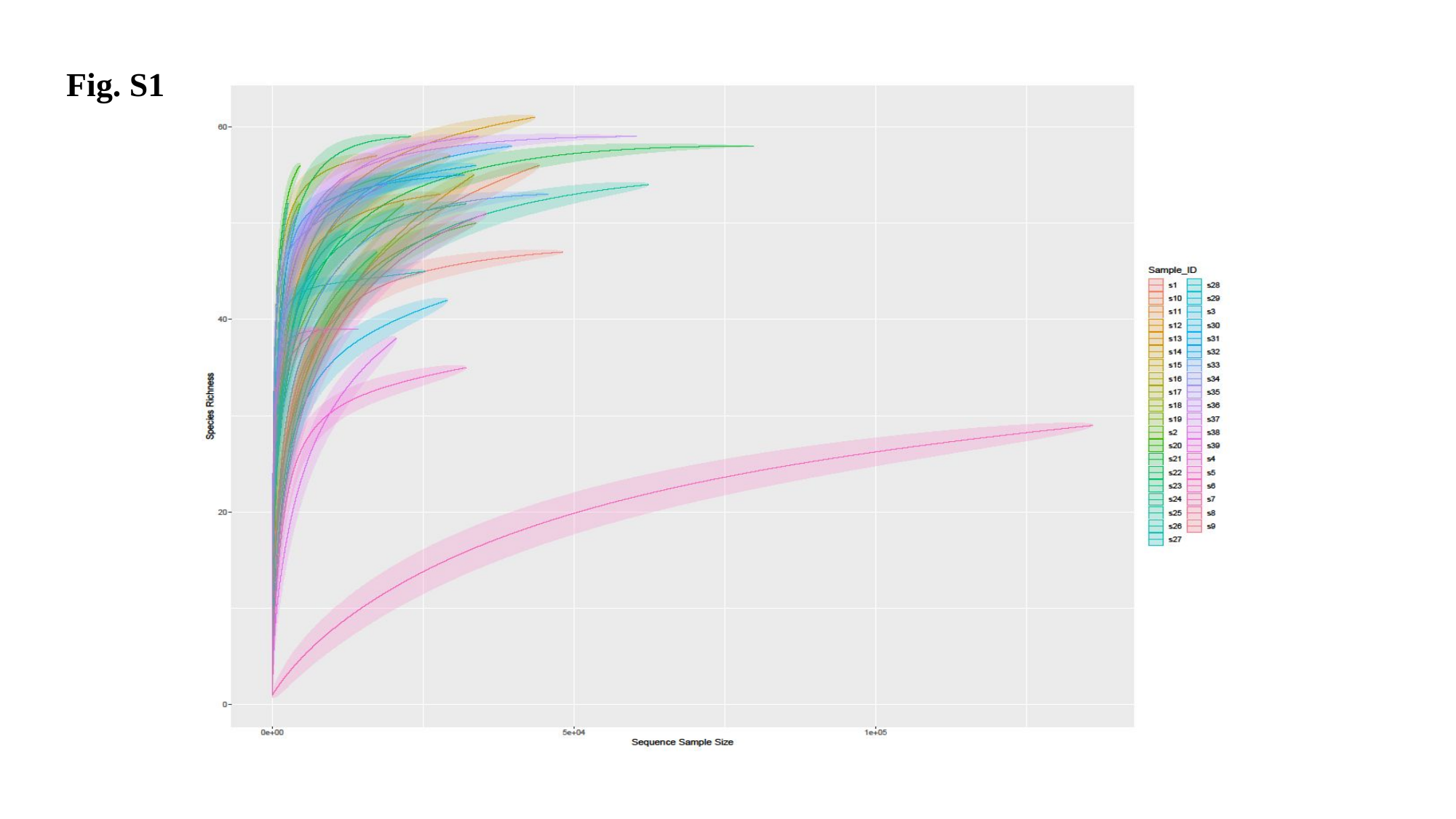

Fig. S1

## Slide 2
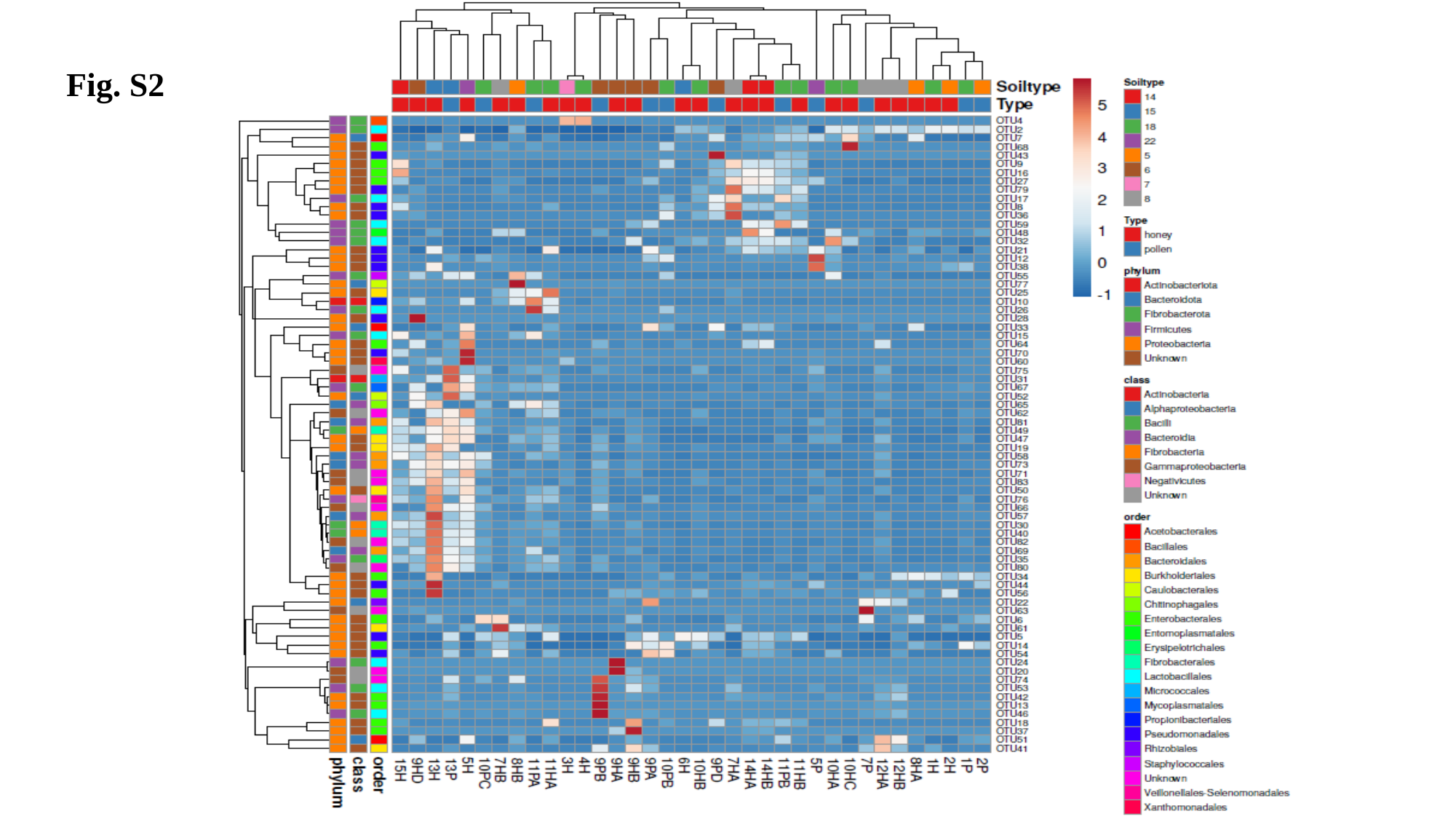

Fig. S2

Supplement: Supplementary file 2 — Additional file 2: Fig S1. Rarefaction curves illustrating the sequencing depth of bacterial communities (identified OTUs) in the 39 samples of honey and pollen collected from the 15 apiaries of this study. Curves were generated in R using the package ggrare and plotted with ggplot2. Fig S2. Clustering of OTUs from our beehive samples provides a proportional representation of microbiomes at 3 taxonomical levels (phylum-class-order) across different samples of honey and pollen and soil type habitats. The IDs for the samples are indicated at the bottom, where the numbers designate the apiary (postcode location) and letters H or P denote the product type -honey or pollen, respectively. If H or P are followed by another letter (A-B-C-D) indicates different beehives within the same apiary. Different soils include: 5, Herb-rich chalk and limestone pastures, lime-rich deciduous woodlands; 6, Neutral and acid pastures and deciduous woodlands, acid communities such as bracken and gorse in the uplands; 7, Base-rich pastures and deciduous woodlands; 8, Wide range of pasture and woodland types; 14, Mostly lowland dry heath communities; 15, Mixed dry and wet lowland heath communities; 18, Grassland and arable some woodland; and 22: Arable grassland and woodland. The heatmap was generated using ClustVis. [file 40793_2023_460_MOESM2_ESM.pptx]
